# AMBtalk: A Cardiovascular IoT Device for Ambulance Applications

**DOI:** 10.3390/s21082781

**Published:** 2021-04-15

**Authors:** Wen-Liang Chen, Yi-Bing Lin, Ted C.-Y. Chang, Yan-Ren Lin

**Affiliations:** 1 Department of Biological Science and Technology, National Yang Ming Chiao Tung University, Hsinchu City 30010, Taiwan; wenurea@gmail.com; 2 College of Artificial Intelligence, National Yang Ming Chiao Tung University, Hsinchu City 30010, Taiwan; 3 Institute of Architecture, China Medical University, Taichung 406, Taiwan; 4 Department of Computer Science and Information Engineering, Asia University, Taichung 41354, Taiwan; 5 Department of Electrical Engineering, National Cheng Kung University, Tainan 701, Taiwan; 6 Quanta Computer Lnc., Taipei 111, Taiwan; ted.chang@quantatw.com; 7 Department of Emergency and Critical Care Medicine of Changhua Christian Hospital, Changhwa County 500, Taiwan; h6213.lac@gmail.com

**Keywords:** acute coronary syndrome (ACS), ambulance, cardiovascular IoT device, emergency medical service (EMS), Electrocardiogram (ECG)

## Abstract

Acute Coronary Syndrome (ACS) and other heart emergency events require immediate chest pain identification in the ambulance. Specifically, early identification and triage is required so that patients with chest pain can be quickly sent to a hospital with appropriate care facilities for treatment. In the traditional approach, ambulance personnel often use symptom checklists to examine the patient and make a quick decision for the target hospital. However, not every hospital has specialist facilities to handle such emergency cases. If the result of the subsequent cardiac enzyme test performed at the target hospital strongly suggests the occurrence of myocardial infarction, the patient may need to be sent to another hospital with specialist facilities, such as Percutaneous Coronary Intervention. The standard procedure is time consuming, which may result in delayed treatment and reduce patent survival rate. To resolve this issue, we propose AMBtalk (Ambulance Talk) for accurate, early ACS identification in an ambulance. AMBtalk provides real-time connection to hospital resources, which reduces the elapsed time for treatment, and therefore, improves the patient survival rate. The key to success for AMBtalk is the development of the AllCheck^®^ Internet of Things (IoT) device, which can accurately and quickly provide cardiovascular parameter values for early ACS identification. The interactions between the AllCheck^®^ IoT device, the emergency medical service center, the ambulance personnel and the hospital are achieved through the AMBtalk IoT server in the cloud network. AllCheck^®^ outperforms the existing cardiovascular IoT device solutions for ambulance applications. The testing results of the AllCheck^®^ device show 99% correlation with the results of the hospital reports. Due to its excellent performance in quick ACS identification, the AllCheck^®^ device was awarded the 17th Taiwan Innovators Award in 2020.

## 1. Introduction

Cardiovascular disease is one of the most serious chronic diseases due to the aging population structure and changes in living patterns and eating habits. The most common type of heart disease is acute coronary syndrome (ACS), which refers to acute myocardial infarction (MI) or hypoxia, and is often caused by a sudden decrease in coronary artery blood flow. MI is a highly prevalent and fatal disease in the world. 

**Figure 1:**
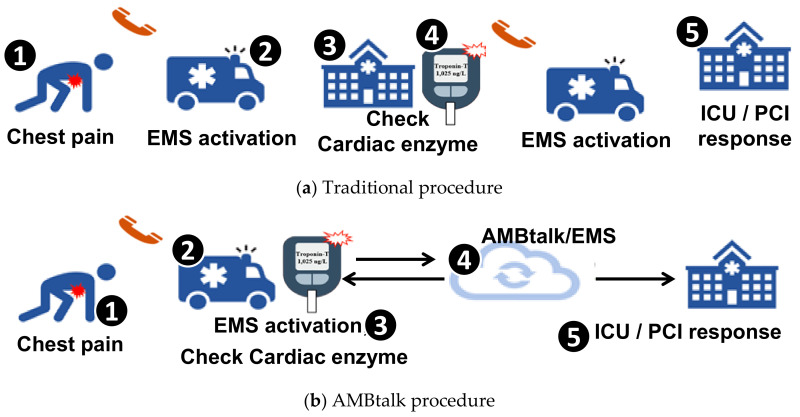
The Emergency Medical Service (EMS) procedures for ambulance; ICU: Intensive Care Unit; PCI: Percutaneous Coronary Intervention.

ACS or other heart emergency events require immediate medical care. Therefore, the rapid diagnosis of MI is a very important topic in the Emergency Medical Service (EMS) center. However, post myocardial-infarction care is accessible only at centralized specialist facilities, and not every hospital has such facilities. Therefore, early identification and triage is required so that the patient with chest pain can be quickly sent to a hospital with acute myocardial-infarction care facilities for treatment. Accurate early identification is a challenging task in the ambulance. Suppose that a patient with chest pain calls for Emergency Medical Service ((1) in Figure 1a). Upon arrival at the patient location ((2) in Figure 1a), the ambulance personnel often use symptom checklists to examine the patient and make a quick decision for an appropriate receiving facility ((3) in Figure 1a). If the result of the cardiac enzyme test in the hospital ((4) in Figure 1a) strongly suggests the occurrence of myocardial infarction, the patient may need to be sent to another hospital with specialist facilities, such as Percutaneous Coronary Intervention (PCI) ((5) in Figure 1a). The above procedure is time consuming, which may result in delayed treatment and may reduce patient survival rate. For example, in Changhua County in Taiwan, there are more than 200 hospitals with various specialist facilities, and the patients are often sent to the wrong hospitals at first. Therefore, it is important that Point-of-Care (POC) testing is made available in the ambulance.

POC medical diagnostic testing is performed at the time and place of patient care, utilizing small biological samples (e.g., blood samples) for rapid diagnosis. Specifically, POC tests must provide rapid detection, ease of use, and high portability [1]. For cardiovascular disease, POC testing of several biomarkers must be performed in the ambulance. According to the National Academy of Clinical Biochemistry, myocardial biomarkers can effectively demonstrate myocardial necrosis in patients with clinical history of MI-related symptoms; specifically, a myocardial biomarker value that exceeds the 99th percentile upper reference limit of the healthy population measured using a device with coefficient of variation (CV) of less than 10% indicates myocardial necrosis. In this paper, the myocardial biomarkers are cardiac troponin I (cTnI), cardiac troponin T (cTnT), creatine phosphokinase (CPK), and creatine kinase-MB (CK-MB). Their normal values are less than 19.8 ng/L (cTnI), 14 ng/L (cTnT), 223 U/L (CPK), and 6.3 ng/L (CK-MB), respectively.

CK-MB is an enzyme that exists in the heart, skeletal muscle, small intestine, etc. A CK-MB to total CPK ratio of 2.5% or more is specific for myocardial injury. The European and US guidelines recommend using cTnI or cTnT to diagnose ACS. However, CK-MB is a stronger predictor of the prognosis of patients with ACS than cTnI and cTnT, and still has important clinical value in patients with MI. Therefore, these four biomarkers play different roles in diagnosis (cTnI/cTnT) and prognosis (CK-MB/CPK) of ACS.

Recently, numerous devices have been developed for the purpose of POC testing. However, the limitations of these devices prevent reliable diagnosis. For example, some ambulances have been equipped with mobile stroke units so that thrombolysis can be delivered more rapidly. Such scenario requires a stroke specialist to be present in the ambulance, which is too expensive and is not a standard emergency care service in many healthcare systems. Similarly, POC testing equipment for myocardial infarction diagnosis also requires trained operators [2], and rapid in-ambulance screening is, therefore, not currently feasible.

Very little research has been devoted to the development of POC devices for the detection of cTnI, cTnT, CPK, and CK-MB. Therefore, rapid and accessible ACS diagnosis in an ambulance remains elusive. In this paper, we propose AMBtalk (Ambulance Talk) for accurate and early ACS identification in an ambulance. AMBtalk provides real time connection to hospital resources, which reduces the elapsed time for treatment, and therefore, improves the patient survival rate. The key to success for AMBtalk is the development of the AllCheck^®^ Internet of Things (IoT) device, which can provide cTnI, cTnT, CPK, and CK-MB values quickly.

The AMBtalk procedure is illustrated in Figure 1b. A patient with chest pain calls for EMS ((1) in Figure 1b). After the ambulance arrives at the patient location ((2) in Figure 1b), the ambulance personnel use AllCheck^®^ to measure the patient’s blood sample. Besides these biomarker levels, electrocardiogram (ECG) readings also have significant implications to a patient’s condition. In this paper, both AllCheck^®^ and an ECG device are used in the ambulance ((3) in Figure 1b). A video for the AMBtalk actions in the ambulance can be found in [3]. The sensor data are sent to the AMBtalk server in the cloud to conduct diagnosis ((4) in Figure 1b). Based on the result of diagnosis, AMBtalk automatically contacts the most available hospital with appropriate specialist facilities, and instructs the ambulance to send the patient to that hospital ((5) in Figure 1b). Several studies provide ambulance dispatching algorithms that can be used by the EMS center. One study [4] provided the fastest service by identifying the nearest ambulance with the shortest route to the patient’s site. Another study [5] proposed a smart ambulance system that keep the road drivers aware of the emergency routes chosen by ambulances to improve the transportation time of patients. These solutions can be used in (1) and (5) of Figure 1b. The details are out of the scope of this paper and can be found in [4,5].

The interactions between the AllCheck^®^ IoT device, the EMS center, the ambulance personnel, and the hospital are achieved through the IoT technologies described in [6] and [7]. This paper is organized as follows: Section 2 elaborates on the AllCheck^®^ IoT device. Section 3 introduces the procedure in the EMS center. Section 4 describes the implementation of AMBtalk in a cloud network. Appendix A provides the acronyms used this paper.

## 2. The AllCheck^®^ IoT Device

Currently, label-free affinity electrochemical biosensors consisting of electrodes and mediators show great promise for POC use [8,9,10]. This section describes AllCheck^®^, a POC IoT device for ACS detection [11]. AllCheck^®^ uses semiconductor manufacturing technology (SMT) to enhance consistency of electrode assembly and a unique biomediator to reduce noise, not only demonstrates high accuracy and reproducibility, but is also portable, requires short reaction time, and shows high potential for POC testing.

We implemented SMT-produced electrodes and a biosynthesized streptavidin mediator that exhibits strong binding affinity for biotinylated bioreceptors. We also designed bioreceptor modification for better biosensor stability. Our label-free affinity detection design results in an easy-to-manufacture and high-accuracy biosensor for a wide range of targets.

The Clinical and Laboratory Standards Institute (CLSI) has defined POC guidelines (EP05-A3, EP24-A2, EP25-A) for reproducibility, accuracy, and stability, where the CV for the measurements must be less than 10%. Since our biosensor directly measures the electrical signal on the SMT chip surface, the accuracy can be adjusted by the electrode production settings to satisfy the POC guidelines. Such settings include the thickness of the thin-film metal and surface roughness [12].

**Figure 2:**
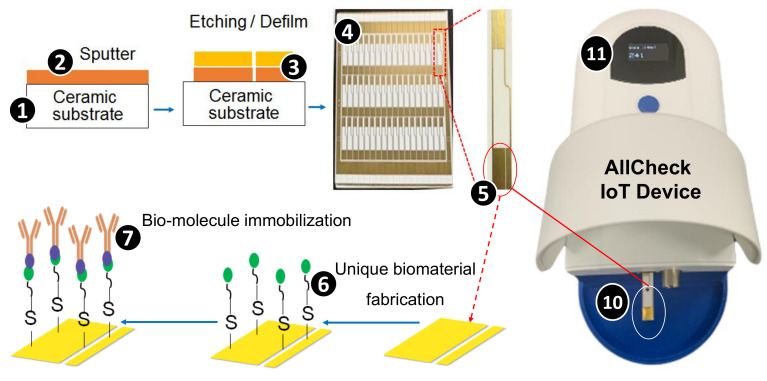
Development of a Point-of-Care (POC) biosensor.

Although the streptavidin biomediator design improves stability, direct immobilization of bioreceptors onto the mediator restricts its orientation, which limits the accuracy. To resolve this issue, we introduced a unique linker with ideal flexibility and rigidity to the streptavidin biomediator [13]. The electrodes were produced using the direct plate copper technology on a ceramic substrate (Figure 2 (1)). We sputtered Cu on the substrate (Figure 2 (2)), created resistance coating, and exposed, developed, and electroplated AU. Then, we etched and defilmed the thin-film electrodes (Figure 2 (3)) by maintaining a thickness greater than 0.1 μm and surface roughness less than 0.3 μm to ensure accuracy. We used X-ray fluorescence spectroscopy to measure the thickness of the samples. The results indicated that the thickness ranges from 0.1 μm to 0.124 μm, with an average of 0.0121 ± 0.0034 μm. An Alpha-Step^®^ D-500 Stylus Profiler was used to evaluate the surface roughness. The results indicated that the surface roughness ranges from 0 to 0.3 μm, with an average of 0.087 ± 0.0124 μm. Therefore, the quality of bare chips (Figure 2 (4)) is highly stable, which is suitable for mass production.

In a unique biomaterial fabrication, we first immersed the reaction area of electrodes (see Figure 2 (5)) in an ethanolic solution of 11-MUA to establish self-assembled monolayers. Then, we conducted the 1-Ethyl-3-(3-dimethylaminopropyl)-carbodiimide (EDC) and N-hydroxysuccinimide (NHS) coupling reaction to activate the carboxyl-terminated group of 11-MUA [14]. We constructed a GW linker (GINSSSVPGDPPW) with the streptavidin sequence as a unique biomaterial (biomediator; Figure 2 (5)) and cloned it into the pET-30a vector for recombinant protein expression and purification. Then, we modified the biomediator onto the chips through the covalent bond linkage by the chemical reaction of activated carboxyl-terminated group with amide-terminated group in the biomediator (Figure 2 (6)). To immobilize the bioreceptor on the electrode, we performed NHS-PEG_4_-biotinylation to label bioreceptors with biotin, and added biotinylated bioreceptors to the surface of the biomediator-modified electrodes (Figure 2 (7)).

The AllCheck^®^ biosensor chips were modified with anti-cTnI, cTnT, CK-MB, and CPK antibodies, respectively. We conducted electrochemical impedance spectroscopy (EIS) measurements to determine the reproducibility and accuracy. Let us take cTnI as an example. The CV of the charge transfer resistance $R_{CT}$ for each chip was used to indicate reproducibility. The accuracy is the difference between the detected values of $R_{CT}$ as transformed into the calculated blood concentrations and the actual values obtained by testing blood samples in the hospital. We measured the stability, namely, the variation of $R_{CT}$ during a period of 9 weeks. Our experiments indicate that AllCheck^®^ possesses accuracy, reproducibility, and stability of 93%, 96%, and 94%, respectively. Therefore, biosensors produced using the combination of SMT-produced electrodes and our unique biomaterial meet the criteria of the CLSI standards for POC testing. More details about our POC biosensor can be found in [11].

In the AllCheck^®^ analysis method, the device uses EIS at fixed frequency with an amplitude of 10 mV to detect target analytes. The frequency is set in the range of 1–10 HZ, depending on the detection performance of different types of biosensor chips. The sensor collects nine impedance readings, which are averaged to obtain the final measured value.

Following the guidelines of European Society of Cardiology (ESC), the specific monitoring biomarker “*X*” in the blood sample for ACS diagnosis are cTnI, cTnT, CPK, and CK-MB. The mean values of the above biomarkers are interpolated using the formula Equation (1) of the standard curve to acquire the concentrations of the target biomarkers.

(1) $$f_{X}\left(x\right)=e^{\left(\frac{a_{0}\times \sqrt{a_{1}\times x+a_{2}}+a_{3}}{a_{4}}\right)}\;\;\;\;\;\;\;\;\;$$

In Equation (1), X is the target biomarker, x is the impedance mean value of target biomarker, and $f_{X}\left(x\right)$ is the concentrations of target biomarkers for X. For X= cTnI, $a_{0}=1$, $a_{1}=1,843,700$, $a_{2}=3,731,450,533$, $a_{3}=49,310$, $a_{4}=18,437$. For X= cTnT, $a_{0}=1$, $a_{1}=59,923,000$, $a_{2}=25,424,176,369$, $a_{3}=157,535$, $a_{4}=59,923$. For X= CPK, $a_{0}=5$, $a_{1}=-14,867$, $a_{2}=371,363,182$, $a_{3}=186,650$, $a_{4}=14,867$. For X= CK-MB, $a_{0}=2$, $a_{1}=2,777,500$, $a_{2}=28,658,679$, $a_{3}=11,085$, $a_{4}=1111$.

**Figure 3:**
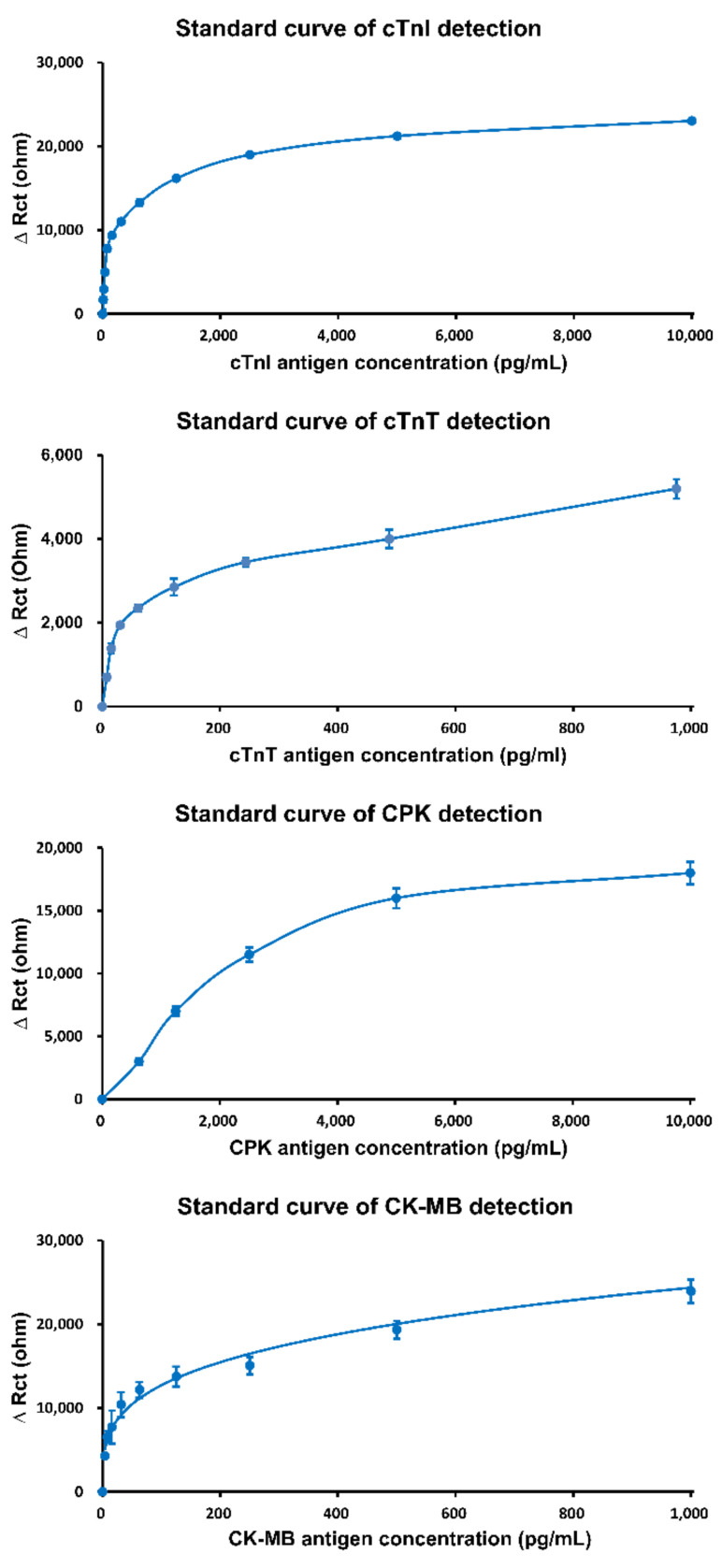
The performance of the AllCheck^®^ device.

Currently, most electrochemical detection is performed using the amperometric method, in which matrix effects of clinical samples easily interfere with detection signals [15]. In contrast, the EIS method (AC impedance) of AllCheck^®^ can analyze the signals at a specific frequency, which avoids the noise (nonspecific signals) of clinical samples. Therefore, the AllCheck^®^ device can be used to analyze whole blood samples directly. In addition, the device has a high quality of reproducibility (>96%), accuracy (>93%), and stability (>94%), which outperforms the existing electrochemical biosensor products. Moreover, the device has high sensitivity (protein detection: 1 pg/mL) and low sample volume requirement (20 μL), with fast detection time (5 min). Figure 3 shows the performance of the AllCheck^®^ device in terms of cTnI, cTnT, CPK, and CK-MB. The figure indicates that the values of these biomarkers can be accurately measured.

To operate the AllCheck^®^ device on the ambulance, the biosensor chip first needs to be inserted into the device (Figure 2 (10)). The blood sample volume of 20 μL should then be dropped on the biosensor chip surface for antibody-antigen reaction. The biosensor chip should next be washed with a wash buffer and dried using a cotton swab. Then, the detection buffer should be dropped on the chip surface, and detection begins when the button is pressed. AllCheck^®^produces the results in 3 min. The results of cardiac biomarkers as well as the ECG data are displayed on the screen of AllCheck^®^ (Figure 2 (11)) and are uploaded to the cloud for diagnosis by hospital staff [3]. The diagnosis result will then be connected with the treatment resources through the AMBtalk system to direct the ambulance to a suitable hospital in real time (to be elaborated in Section 4). In this way, AMBtalk is able to send the patient to the specific hospital rapidly to reduce waiting for treatment and improve patient survival.

Several commercial devices have been designed to detect cardiac biomarkers in blood samples (the information regarding these were obtained from their official websites). The Abbott i-stat test system^®^ is portable, with a 10-min testing time using blood samples of 2–3 mL. The Roche Cobas h 232 POC system^®^ is portable, with a 15-min testing time using blood samples of 2–3 mL. The Beckman coulter immunoassay system^®^ is not portable, with a 35-min testing time using blood samples of 3–5 mL. The Roche Cobas analyzer for immunoassay^®^ is not portable, with a 25-min testing time using blood samples of 3–5 mL. These devices must be operated by medical technologist, physicians, or nurses in hospital emergency rooms or mini laboratories. On the other hand, AllCheck^®^ is portable, with 5-min testing time using a blood sample of 20 μL. AllCheck^®^ is designed for operation by staff in the ambulance and diagnosis can be performed remotely by the medical technologist, physician, or nurse. AllCheck^®^ can also be operated in emergency rooms.

To prove the reliability, AllCheck^®^ has received permission for testing human clinical samples by the Institutional Review Board (IRB), and the required documents from the Taiwan Food and Drug Administration (TFDA) have been prepared for acquiring the certificate. The testing results of the AllCheck^®^ device show 99% correlation with the results of the hospital report. Overall, the results mentioned above verify the integrity of the AllCheck^®^ device.

## 3. The Procedure in the EMS Center

According to the recommendations of the American Heart Association Minnesota Emergency department (ED) Chest Pain Protocol, if patients with chest pain come to the ED, the following guidelines of the Minnesota ED chest pain protocol should be followed. The first step involves undergoing examination of a 12-lead ECG and testing of myocardial biomarkers in the blood. From the ECG result, physicians will determine whether it is ST-segment elevation myocardial infarction (STEMI). STEMI is defined as an ECG wave pattern elevate more than 1 mm in the ST segment with more than two contiguous leads, which means that the patient’s cardiomyocytes have a large number of necrosis and must be treated with cardiac catheterization immediately.

If the patient does not have STEMI, physicians should wait for the report of myocardial biomarkers to evaluate the patient. In the Changhua Christian Hospital, patients with cTnI or cTnT values greater than 19.8 ng/L or 14 ng/L, respectively, are considered at high risk of ACS, and the ambulance must deliver them to the hospital with PCI equipment. In addition, CK-MB and CPK are also tested, as the CK-MB/CPK ratio is a strong predictor of the prognosis of patients with MI, with a higher ratio indicating higher risk.

While waiting for the myocardial biomarker results, the ECG should be repeated immediately when the patient’s chest pain changes. If the ECG readings change to indicate STEMI, the patient will be treated with cardiac catheterization. When the first myocardial biomarker results are observed, the heart score will be calculated. Heart score, which is calculated using ECG readings, biomarker levels, and other characteristics of the patient, such as age, are used to assess the risk of myocardial infarction in the patient, and with it, patients can be classified into low risk, moderate risk, or high risk. A heart score of 0–3 with negative myocardial biomarker results means that the patient belongs to the low-risk group, and the second ECG and myocardial biomarker testing will be performed in the ED after 2–3 h. A heart score of 4–6 with negative myocardial biomarker results indicate moderate risk, and the patient should be hospitalized or placed in the observation unit to have serial ECGs monitored in addition to being tested for myocardial biomarkers at the third and sixth hours. Lastly, belonging to the high-risk group is determined by a patient heart score of 7–10 or a positive myocardial biomarker result, and thus, should be diagnosed with non-ST segment elevation myocardial infarction (NSTEMI) and must be hospitalized or treated in the Intensive Care Unit (ICU) immediately.

The American Heart Association Minnesota ED Chest Pain Protocol is used in Taiwan, which is shown in Step 4 of Figure 1b. Specifically, based on the result obtained from the AllCheck^®^ device, we use this protocol for pain chest to automatically decide what medical facility is needed for the patient. The EMS center then maps the selected medical facility to appropriate hospitals, and provides a target hospital list to the ambulance personnel. The EMS center can also provide optimal routes through the algorithms proposed in [4,5].

## 4. Cloud-Based AMBtalk Network Architecture

**Figure 4:**
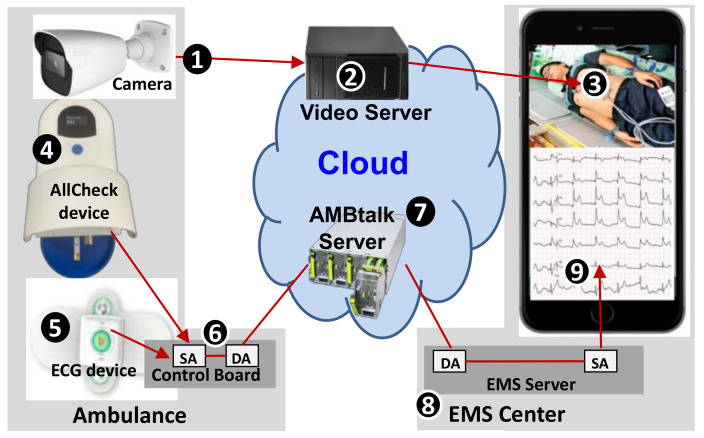
The cloud-based AMBtalk system.

AMBtalk is an application of an IoT development platform called IoTtalk [6]. With AMBtalk, a camera is installed in the ambulance (Figure 4 (1)) so that the EMS center can monitor the patient on the big screen of the Integrated Operation Center (IOC) or the on a smartphone (Figure 4 (3)) through a video server (Figure 4 (2)). The AllCheck^®^ device (Figure 4 (4)) and the ECG device (Figure 4 (5)) sends out the measured data to the AMBtalk server (Figure 4 (7)) through a control board in the ambulance (Figure 4 (6)). The AMBtalk server then sends these data to the EMS center (Figure 4 (8)) for diagnosis. The data can be shown on the big screen of the IOC or on any smartphone (Figure 4 (9)). The AMBtalk server is deployed at the EMS center or in the cloud, and is connected to the EMS server through Ethernet wired communications. An ambulance control board is installed in the ambulance and is connected to the AMBtalk server through 4G LTE or 5G wireless communications.

In the control board of the ambulance (Figure 4 (6)), there are two software modules: Device Application (DA) and Sensor and Actuator Application (SA). The DA is responsible for communication with the AMBtalk server, which is implemented using HTTPs or MQTT.

The SA of the ambulance control board produces two datasets: the cardiovascular sensor values (cTnI, cTnT, CPK and CK-MB) measured from the AllCheck^®^ device (Figure 4 (4)) and the 12-lead ECG signals measured from the ECG device (Figure 4 (5)). The SA for the AllCheck^®^ device in the ambulance control board (Figure 4 (4)) computes the cardiovascular sensor values, i.e., the concentrations $f_{X}\left(x\right)\;$of target biomarker “*X*” in the blood sample, where *X* = cTnI, cTnT, CPK, and CK-MB in Equation (1). The SA algorithm in the ECG device captures the pattern of the ST segment of ECG readings. Both the cardiovascular sensor and the ECG data are used by the SA to determine whether the patient has STEMI or not.

On the EMS server, SA and DA software modules are installed, just like the control board in the ambulance. The DA is responsible for the communication between the EMS center and the AMBtalk server. Based on the cardiovascular sensor values and the ECG signals received from the AMBtalk server, the SA of the EMS server (Figure 4 (8)) uses the procedure described in Section 3 to determine if the patient needs PCI, ICU, or ED treatment. Specifically, a new ST segment elevation in at least two contiguous leads of greater than or equal to 1 mm indicates cardiomyocyte necrosis in the patient, which requires immediate cardiac catheterization. The ECG dashboard displays the measured data and the diagnosis results (Figure 4 (9)). The SA also uses the algorithms described in [4,5] to select an appropriate hospital. The EMS center sends the hospital name to the ambulance through the connection to the AMBtalk server.

**Figure 5:**
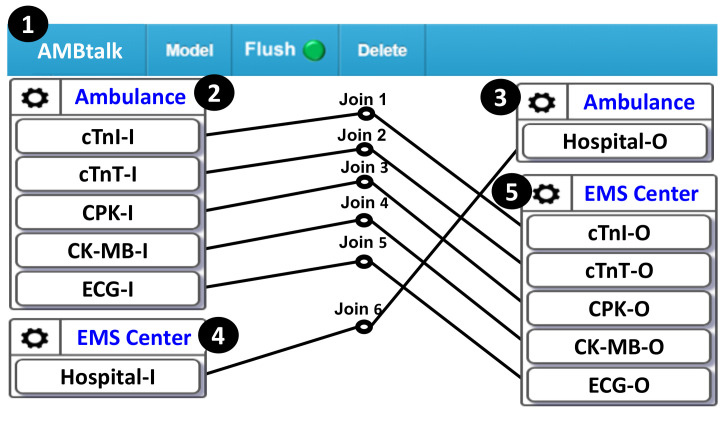
The AMBtalk graphical user interface.

The AMBtalk server is responsible for processing the data received from the control board of the ambulance and forwards them to the EMS server. Such a task is easily achieved by the settings in the AMBtalk Graphical User Interface (GUI; Figure 5 (1)). In this GUI, the “Ambulance” icon (Figure 5 (2)) represents the control board in the ambulance (Figure 4 (6)), which receives the measured cardiovascular and the ECG data from the control board and puts them in five variables, i.e., cTnI-I, cTnT-I, CPK-I, CK-MB-I, and ECG-I, to be sent to the AMBtalk server. Another icon “EMS Center” (Figure 5 (5)) represents the EMS server (Figure 4 (8)), which receives the cardiovascular sensor values and the ECG signals from the AMBtalk server through the variables cTnI-O, cTnT-O, CPK-O, CK-MB-O, and ECG-O. Therefore, to forward the data from the ambulance to the EMS center, the lines simply need to be dragged to connect the icons in (Figure 5 (2)) to the icons in (Figure 5 (5)), e.g., see the lines “Joins 1–5.”

Similarly, after the EMS server has determined the target hospital for the patient based on the received sensor data, the AMBtalk server forwards the decision to the ambulance. This task is achieved by creating Hospital-I (Figure 5 (4)) in the EMS server SA and Hospital-O (Figure 5 (3)) in the control board SA of the ambulance. Then, the user needs to drag the Join-6 line ((4)→(3) in Figure 5) to create the data path. Based on the above description, it is clear that the ambulance application can be easily implemented in the AMBtalk GUI.

## 5. Conclusions

In Taiwan, heart disease ranks second among the top 10 causes of death [16]. In this paper, we proposed AMBtalk (Ambulance Talk) for accurate early ACS identification in the ambulance. AMBtalk provides real time connection to hospital resources, which reduces the elapsed time for treatment, and therefore, improves patient survival rate. The key to success for AMBtalk is the development of the AllCheck^®^ IoT device, which can accurately and quickly provide cTnI, cTnT, CPK, and CK-MB values for early ACS identification. The interactions between the AllCheck^®^ IoT device, the emergency medical service center, the ambulance personnel, and the hospital are achieved through the AMBtalk IoT server in a cloud network.

We showed that AllCheck^®^ outperforms the existing cardiovascular IoT device solutions for ambulances. The best existing solution requires a 10-min testing time using blood samples of 2–3 mL, while AllCheck^®^ produces results in 3 min using a blood sample of 20 μL. The existing solutions must be operated by medical technologists, physicians, or nurses. On the other hand, AllCheck^®^ is designed for operation by staff in the ambulance and diagnosis is performed remotely by the medical technologist, physician, or nurse.

From the data we collected, without early identification of ACS, 15% of the ambulance missions were targeted to the wrong hospitals. With AMBtalk, the ambulances have always sent the patients to the appropriate hospitals.

The price of AllCheck^®^ is USD 500. AMBtalk is an application of Quanta’s medical AI platform QOCA^®^ AIM. The platform is already available in smart hospitals for other purposes, such as smart ward, smart ICU, and so on. There is no extra overhead incurred for the AMBtalk application.

## Data Availability

Data will be provided on request through the first author of this article.

## Appendix A. Acronyms

This appendix provides the acronyms used this paper.

ACS	Acute Coronary Syndrome
AMBtalk	Ambulance Talk
CK-MB	Creatine kinase-MB
CLSI	Clinical and Laboratory Standards Institute
CPK	Creatine phosphokinase
cTnI	Cardiac troponin I
cTnT	Cardiac troponin T
CV	Coefficient of Variation
DA	Device Application
ECG	Electrocardiogram
ED	Emergency department
EDC	-Ethyl-3-(3-dimethylaminopropyl)-carbodiimide
EIS	Electrochemical Impedance Spectroscopy
EMS	Emergency Medical Service
ESC	European Society of Cardiology
GUI	Graphical User Interface
ICU	Intensive Care Unit
IOC	Integrated Operation Center
IoT	Internet of Things
IRB	Institutional Review Board
MI	Myocardial Infarction
NHS	N-hydroxysuccinimide
PCI	Percutaneous Coronary Intervention
POC	Point-of-Care
SA	Sensor and Actuator Application
SMT	Semiconductor Manufacturing Technology
STEMI	ST-segment elevation myocardial infarction
TFDA	Taiwan Food and Drug Administration
